# Ecological momentary assessment versus retrospective assessment for measuring change in health-related quality of life following cardiac intervention

**DOI:** 10.1186/s41687-020-00261-2

**Published:** 2020-11-16

**Authors:** Tom H. Oreel, Philippe Delespaul, Iris D. Hartog, José P. S. Henriques, Justine E. Netjes, Alexander B. A. Vonk, Jorrit Lemkes, Michael Scherer-Rath, Hanneke W. M. van Laarhoven, Mirjam A. G. Sprangers, Pythia T. Nieuwkerk

**Affiliations:** 1 Department of Medical Psychology, Amsterdam University Medical Centers, Location Academic Medical Center, University of Amsterdam, Amsterdam Public Health Research Institute, Meibergdreef 15, J3-212, Amsterdam, 1105 AZ The Netherlands; 2 Department of Psychiatry and Neuropsychology, Maastricht University Medical Center, School for Mental Health and Neuroscience, Vijverdalseweg 1, Maastricht, 6200 MD The Netherlands; 3 Mondriaan Mental Health Care, John F. Kennedylaan 301, Heerlen/Maastricht, 6419 XZ The Netherlands; 4 Faculty of Philosophy, Theology and Religious Studies, Radboud University Nijmegen, Erasmusplein 1, Nijmegen, 6525 HT The Netherlands; 5 Department of Cardiology, Amsterdam University Medical Centers, Location Academic Medical Center, University of Amsterdam, Meibergdreef 9, Amsterdam, 1105 AZ The Netherlands; 6 Department of Cardio-Thoracic Surgery, Amsterdam University Medical Centers, Location VU University Amsterdam, De Boelelaan 1117, Amsterdam, 1081 HV The Netherlands; 7 Department of Cardiology, Amsterdam University Medical Centers, Location VU University Amsterdam, De Boelelaan 1117, Amsterdam, 1081 HV The Netherlands; 8 Department of Medical Oncology, Cancer Center Amsterdam, Amsterdam University Medical Centers, University of Amsterdam, Meibergdreef 9, Amsterdam, 1105 AZ The Netherlands

**Keywords:** Health-related quality of life, Ecological momentary assessment, Retrospective assessment, Validity, Cardiac intervention

## Abstract

**Background:**

Measuring change in health-related quality-of-life (HRQoL) is important to assess the impact of disease and/or treatment. Ecological momentary assessment (EMA) comprises the repeated assessment of momentary HRQoL in the natural environment and is particularly suited to capture daily experiences. Our objective was to study whether change in momentary measures or retrospective measures of HRQoL are more strongly associated with criterion measures of change in HRQoL.

Twenty-six coronary artery disease patients completed momentary and retrospective HRQoL questionnaires before and after coronary revascularization. Momentary HRQoL was assessed with 14 items which were repeatedly presented 9 times a day for 7 consecutive days. Each momentary assessment period was followed by a retrospective HRQoL questionnaire that used the same items, albeit phrased in the past tense and employing a one-week time frame. Criterion measures of change comprised the New York Heart Association functioning classification system and the Subjective Significance Change Questionnaire. Regression analysis was used to determine the association of momentary and retrospective HRQoL change with the criterion measures of change.

**Results:**

Change according to momentary HRQoL items was more strongly associated with criterion measures of change than change according to retrospective HRQoL items. Five of 14 momentary items were significantly associated with the criterion measures. One association was found for the retrospective items, however, in the unexpected direction.

**Conclusion:**

Momentary HRQoL measures better captured change in HRQoL after cardiac intervention than retrospective HRQoL measures. EMA is a valuable expansion of the armamentarium of psychometrically sound HRQoL measures.

**Supplementary Information:**

The online version contains supplementary material available at 10.1186/s41687-020-00261-2.

## Background

Nowadays, health-related quality of life (HRQoL) is considered an important outcome in healthcare research. HRQoL is typically assessed using self-report questionnaires in which patients are asked to make assessments over the past week(s) or month(s). These reports require patients to recollect past experiences and combine that information to respond to a questionnaire item, thereby providing a global evaluation of their past HRQoL. For example, item 32 of the Short Form Health Survey [1] reads: “During the past 4 weeks, how much of the time has your physical health or emotional problems interfered with your social activities (like visiting with friends, relatives, etc.)?”. To answer such items, patients typically need to remember specific situations over a four-week period and be able to reactivate how physical health and/or emotional problems interfered with these (social) activities. Even when all the information can be retrieved, widely diverse experiences must be combined in a summarizing score ranging from 1 (“all of the time”) to 5 (“none of the time”). Such retrospective HRQoL measures may therefore be limited by the individuals’ ability to recall past experiences related to their HRQoL, i.e., they are subject to recall bias [2, 3]. Other cognitive biases also play a role, such as the tendency to judge past experiences by the most intense and/or recent experience rather than the average of all experiences (peak-end rule) [4, 5].

Alternatively, one can assess HRQoL using ecological momentary assessment (EMA). EMA comprises the repeated assessment of patients’ momentary HRQoL in their natural environment [6–8]. Such momentarily assessed HRQoL captures patients’ real-time experiences and can be related to disease and/or treatment [9, 10]. Because EMA assesses patients’ currently experienced HRQoL, it might be less susceptible to recall and other cognitive biases [8]. However, a disadvantage of EMA is that it is time consuming, burdensome and intrusive to patients due to the need for frequent and repeated assessments [11].

EMA has repeatedly been proposed to assess HRQoL in patients with mental health problems [6, 7, 12, 13] but has not often been applied in patients with a chronic illness of a predominant somatic nature, such as cardiovascular disease. Consequently, there is only limited evidence for the reliability and validity of measuring change in HRQoL in somatic diseases using EMA. Measuring change in HRQoL is particularly important to assess the consequences of disease and/or treatment. Currently, it is unknown whether retrospective or momentary measures of HRQoL are equally associated with criterion measures of change and thus yield the same levels of criterion validity.

Our objective was to study whether change in momentary measures or retrospective measures of HRQoL are more strongly associated with criterion measures of change in HRQoL. The objective was examined among patients with coronary artery disease (CAD) who received cardiac revascularization. Patients with CAD typically experience symptoms, such as, chest pain, shortness of breath, and fatigue, which may limit their daily functioning and decrease their HRQoL [14]. Cardiac revascularization procedures aim to alleviate these symptoms and to improve patient’s HRQoL [15, 16]. This patient group was particularly suited for our objective as we expected to find significant changes in HRQoL following this medical intervention.

## Methods

### Study

Patients were enrolled in the multi-center IMPACT study [17, 18], investigating CAD patients with multiple comorbidities. Patients were recruited at the cardiology departments of the Amsterdam University Medical Centers (Amsterdam UMC): Academic Medical Center (AMC) and VU Medical Center (VUmc). The overall objective of the IMPACT study was to improve the conceptualization of HRQoL and to enhance the sensitivity and comprehensiveness of its measurement. A subsample of these patients was enrolled in an add-on study on momentary assessments.

### Patients

Patients were scheduled for cardiac revascularization procedures after being discussed in the multidisciplinary “heart teams”. Patients were eligible if they were 18 years or older, had stable CAD and were scheduled for elective coronary artery bypass graft (CABG) or elective percutaneous coronary intervention (PCI). Patients had to have at least one somatic comorbidity (e.g., diabetes mellitus, obesity, joint disease). Additional inclusion criteria for participation in the current add-on study on momentary assessments were being experienced smart phone users (indicated at their own discretion), and having a functional Wi-Fi connection at home to enable daily transfer of data to a central server to avoid data loss. Patients with cognitive impairments due to brain haemorrhage, cerebral infarction, mental retardation, dementia, Alzheimer’s disease, or patients who were unable to complete questionnaires due language problems were excluded.

### Procedure

**Fig. 1:**
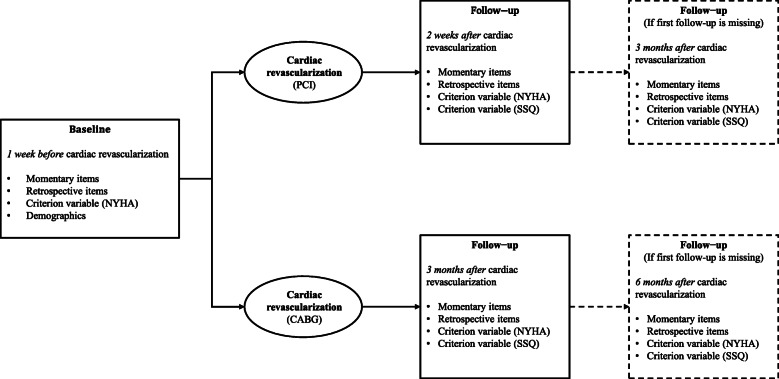
Timeline of the study. *Note.* We only used the data of two time points: *baseline* and *2 weeks* for PCI and *baseline* and *3 months* for CABG. If these follow-up data were missing, data collected at *3 months* (for PCI) and *6 months* (for CABG) were used instead (see dotted line). *NYHA* = New York Heart Association functioning classification system [19, 20], *SSQ* = Subjective Significance Questionnaire [21]

Patients completed momentary and retrospective questionnaires at *baseline* (up to 1 week prior to PCI and CABG) and at two *follow-up* time points. These latter time points differed per vascularization type due to expected difference in recovery, i.e., 2 weeks (for PCI), 3 months (for PCI and CABG), and 6 months (for CABG) following revascularization (see Fig. 1). For this study, we used the data of two time points: *baseline* and *2 weeks* for PCI and *baseline* and *3 months* for CABG. If these follow-up data were missing, data collected at *3 months* (for PCI) and *6 months* (for CABG) were used (see dotted line in Fig. 1). Momentary assessments were conducted over the course of 7 days. Patients received an iPod for the duration of the assessment period with the PsyMate™ application installed (www.psymate.eu). PsyMate™ was programmed to give nine beeps during daytime, at random moments within predefined time slots (maximally 2 h apart). After each beep, a set of items assessing HRQoL was presented. If patients did not respond within 15 min, the application was programmed to close, making response to that particular beep impossible. Hence, the maximum number of completed momentary questionnaires per time period is 63 (seven beeps × 9 days). We did not adopt a minimum completion rate for the momentary questionnaires. Retrospective questionnaires of HRQoL were administered 1 day after completion of the momentary assessments. Since these questions employed a one-week time frame, the period of momentary assessments coincided with that week. Patients had the choice between completing the retrospective questionnaires on paper or online. Criterion measures of HRQoL change were collected together with the retrospective questionnaires at baseline and follow-up. Demographic information was collected at baseline. As the central ethics committee decided that the Medical Research Involving Human Subjects Act did not apply, the study was exempted from further ethical assessment. Written informed consent was obtained from all patients.

### HRQoL measures

#### Momentary items

Patients rated a total of 14 items measuring five dimensions of HRQoL. Items were based on an earlier version developed by Maes and colleagues [9] for patients with tinnitus. We adapted this version to make it more suitable for patients with CAD. We particularly replaced the symptoms of tinnitus by the symptoms CAD patients may have. The *Positive mood* dimension was measured with four items (i.e., “I feel...‘cheerful, ‘relaxed’, ‘energetic, and ‘happy”’). *Negative mood* was measured with four items (i.e., “I feel … ‘anxious’, ‘sad, ‘irritated, and ‘worried”). *CAD symptoms* were measured with five items (i.e., “I feel … ‘tired’, ‘shortness of breath’, ‘pain on my chest’, ‘tightness on my chest’, and ‘an oppressive feeling on my chest”’). *Fatigue* was measured with two items (i.e., “I feel … ‘tired’ and ‘energetic’”). *Pain* was measured with two items (i.e., “I feel … ‘pain’, and ‘pain on my chest”’). All items were rated on a 7-point scale, ranging from 1 (“not at all”) to 7 (“very much”).

#### Retrospective items

Patients rated the same 14 items that were administered as momentary items, now phrased in the past tense, referring to the past week. For example, “past week I felt...‘energetic’, ‘relaxed’, ‘cheerful’, and ‘happy”’. These items were also rated on a 7-point scale, ranging from 1 (“not at all”) to 7 (“very much”). The items were again combined to form the same five dimensions as for EMA: *positive mood, negative mood, CAD symptoms, fatigue* and *pain.*

### Criterion variables

#### Subjective change in HRQoL

Subjective change in HRQoL was measured with the Subjective Significance Questionnaire (SSQ) [21]*.* The SSQ consists of six Likert items measuring subjective change in HRQoL since the cardiac revascularization. From the SSQ we selected three items which provide a criterion measure for the *change in positive and negative mood (*e.g., “To what extent did your emotional state change since the cardiac revascularization?”*), change in fatigue (*e.g., “To what extent did your fatigue change since the cardiac revascularization?”*),* and *change in pain (*e.g., “To what extent did your pain change since the cardiac revascularization?”*).* All items were rated on a 7-point scale, ranging from 1 (“much worse”), to 7 (“much better”). Scores of 1 to 3 represent a decline in HRQoL, 4 no change, and 5 to 7 an improvement in HRQoL since the cardiac revascularization.

#### NYHA class

The NYHA class was measured by the patient-based version of the New York Heart Association (NYHA) functioning classification system [19, 20]. The NYHA classifies functional limitations due to CAD symptoms. NYHA consists of one item assessing limitations during physical activity (I = “not limited in physical activities”, II = “somewhat limited in physical activities”, III = “fairly limited in physical activities”, IV = “not capable of physical activities”). Change in NYHA class provides a criterion measure for the *change in CAD symptoms.*

For assessing changes in pain and fatigue, both the SSQ and NYHA class were used as criterion measures. The separate criterion measures for fatigue (SSQ fatigue) and pain (SSQ pain) coincide conceptually with the pain and fatigue scales. Furthermore, the criterion measure for CAD symptoms (NYHA class) coincide partially with the pain and fatigue scale; i.e., ‘pain on the chest’ and ‘tired’ are considered CAD symptoms. We therefore wanted to examine how these two items relate to the NYHA class.

### Analysis

To enable the comparison of the momentary data with the retrospective data the momentary HRQoL data were combined into an aggregated mean score per assessment period, i.e., an aggregated mean score at baseline and at follow-up. We thereby brought the nested momentary data at the same person level as the retrospective data. This enabled us to analyse the associations with the criterion variables using the same regression model.

#### Scale structures of momentary and retrospective HRQoL measures

Exploratory factor analysis (EFA) was applied to momentary and retrospective items (at baseline and follow-up) to examine the scale structure (positive mood, negative mood, CAD symptoms, fatigue and pain), and whether the scale structure remained stable over time. In all cases we extracted a five factor solution using the minimum residual and varimax rotation method. Furthermore*,* we calculated the Cronbach’s alpha of each scale (momentary and retrospective) at baseline and follow-up. If the expected scale structure was not observed, and/or the reliabilities were suboptimal (Cronbach’s alpha < 0.70), analysis would be performed at item-level.

#### Change in momentary, retrospective HRQoL and criterion measures

For each patient, we first calculated change scores (from baseline to follow-up) for momentary, retrospective and criterion measures (only for change in NYHA class).

##### Change in momentary and retrospective HRQoL

Momentary change scores were calculated by subtracting the average item scores at follow-up from the average item scores at baseline. Retrospective change scores were calculated by subtracting item scores at follow-up from item scores at baseline. A positive change score on the positive mood items (i.e., cheerful, relaxed, energetic and happy) indicates more positive mood. Change scores of the negative mood and CAD symptoms items were reversed, such that a positive change score on negative mood, pain, fatigue, and CAD symptoms also indicates better functioning, i.e., less negative mood and symptomatology.

##### Change in NYHA class

was calculated by subtracting the NYHA class of the follow-up from the NYHA class of the baseline. A positive change score indicates improved physical functioning (less limited in physical activity due to CAD symptoms).

#### Association momentary and retrospective change with criterion measures of change

For each HRQoL outcome, we describe mean momentary and retrospective change for patients who reported declined, unchanged or improved HRQoL on the corresponding criterion measure of change to obtain more insight in the direction and magnitude of change.

For each HRQoL outcome we fitted a separate regression model with momentary and retrospective change as independent variables, and the corresponding criterion measure of change as dependent variable to determine the relative strength of the association of momentary and retrospective change with their corresponding criterion measure of change. For each model we expected a positive association between momentary and retrospective change and the criterion measures of change. Additionally, hierarchical regression analysis was used to determine whether momentary change was significantly more related to the criterion measure than retrospective change. For each HRQoL measure we first fitted a simple regression model with only retrospective change as independent variable. Next, we compared the model fit, using the chi-squared statistic, of the simple model with the full regression model (both momentary and retrospective as independent variables). If the full model fitted significantly better than the simple model, momentary change was significantly more related to the criterion than retrospective change. All regression models were adjusted for baseline momentary (mean) and retrospective scores. Results were considered statistically significant with a 2-sided *p*-value of < 0.05.

#### Software

All analyses were performed in R version 3.6.1 [22].

## Results

### Availability of data and materials

All data analysed for this study are included as supplementary information files.

### Patient characteristics

Data collection took place from 2016 until 2018 (see Table 1 for the patient characteristics). From the 37 patients who agreed to participate in this study, 26 patients (70%) completed both the momentary, retrospective and criterion measures at baseline and at least one follow-up, and were thus included in the present analysis. Of the 11 patients who were excluded from the study, four and three patients did not have momentary measures at baseline and follow-up, respectively, whereas two patients did not provide retrospective measures at baseline and follow-up (see supplementary Table S1).

**Table 1:** Patient characteristics at baseline (*N* = 26)

Characteristics	
Mean age (SD)	68 (7.86)
Number of males (%)	20 (77%)
Cardiac procedure:	
PCI and CAG^a^ (%)	21 (81%)
CABG (%)	5 (19%)
Mean number of momentary observations at baseline (SD)	46 (16.95)
Mean number of momentary observations at follow-up (SD)	50 (15.30)

^a^One patient scheduled for PCI had undergone coronary angiography (CAG) instead

### Momentary versus retrospective change

#### Scale structure

The EFA on momentary items confirmed the scale structure of positive mood, negative mood, CAD symptoms, but not of fatigue and pain, at baseline as well as follow-up (see supplementary Tables S4 and S5). However, the EFA on retrospective items did not confirm any of the scales at both baseline and follow-up (see supplementary Tables S6 and S7). The Cronbach’s alpha coefficients of momentary scales were all above 0.70, except for pain at follow-up. The Cronbach’s alphas of all retrospective scales at baseline and two scales at follow-up were below 0.70 (see supplementary Table S3). Consequently, the subsequent analyses were conducted at item-level.

#### Change in HRQoL

All momentary items indicated an improvement in HRQoL (more positive mood, less negative mood and less symptomology), with the exception of ‘pain’ that increased. Most retrospective items indicated an improvement in HRQoL, however, the items ‘happy’, ‘anxious’, ‘sad’, and ‘irritated’ indicated a decline in HRQoL (see supplementary Table S2).

#### Associations with criterion measures of change

For each HRQoL item, the mean momentary and retrospective change among patients who reported declined, unchanged or improved HRQL on the corresponding criterion measure of change are shown in Table 2. The associations between changes in momentary and retrospective items with the corresponding criterion measures of change, expressed in standardized beta coefficients with standard errors and *p*-values, are given in Table 2.

**Table 2:** Associations between changes in momentary and retrospective items with the criterion measures of change (*N* = 26 at baseline and follow-up)

Momentary and retrospective items	Average change on momentary and retrospective items for different levels of change in corresponding criterion variables			Regression model			Hierarchical regression analysis	
				β	SE	***P*** value	∆F	***P*** value
	**SSQ mood**							
	Decline (*N* = 7)	Same (*N* = 10)	Improve (*N* = 9)					
Cheerful							11.72	<.001*
Momentary	−0.22	− 0.01	0.98	0.80	0.16	<.001*		
Retrospective	0.43	0.40	1.11	0.12	0.19	.557		
Relaxed							2.33	.107
Momentary	0.24	0.20	0.89	0.42	0.26	.124		
Retrospective	0.86	0.90	1.89	0.13	0.23	.583		
Energetic							20.71	<.001*
Momentary	0.30	0.16	1.27	0.99	0.13	<.001*		
Retrospective	0.57	−0.30	0.33	−0.45	0.13	.002*		
Happy							5.01	.010*
Momentary	− 0.09	0.22	1.02	0.70	0.22	.005*		
Retrospective	0.43	−0.60	−0.22	0.03	0.21	.902		
Anxious							2.34	.534
Momentary	0.51	0.02	0.62	0.14	0.78	.859		
Retrospective	−0.29	−0.60	0.22	−0.12	0.26	.645		
Sad							2.10	.134
Momentary	0.23	−0.14	0.59	0.18	0.37	.625		
Retrospective	1.71	−0.20	−1.22	− 0.32	0.24	.192		
Irritated							0.82	.502
Momentary	−0.03	− 0.14	0.59	0.19	0.50	.640		
Retrospective	−0.57	−0.50	− 0.89	− 0.01	0.23	.978		
Worried							2.26	.114
Momentary	0.32	0.12	0.57	0.08	0.38	.839		
Retrospective	1.43	−0.30	0.11	−0.19	0.25	.450		
	**NYHA change**							
	Decline (*N* = 3)	Same (*N* = 7)	Improve (*N* = 19)					
Tired							2.47	.093
Momentary	−0.61	− 0.02	1.32	0.54	0.22	.027*		
Retrospective	1.33	1.57	0.94	0.03	0.26	.923		
Pain chest							4.43	.016*
Momentary	0.03	0.01	0.76	0.04	0.53	.946		
Retrospective	−0.00	−0.57	0.38	0.15	0.26	.557		
Shortness of breath							4.70	.013*
Momentary	−1.83	−0.19	1.50	0.73	0.22	.004*		
Retrospective	1.00	1.43	0.31	−0.34	0.26	0.201		
Tight feeling chest							1.70	.202
Momentary	−0.09	−0.12	1.04	0.44	0.37	.254		
Retrospective	−1.33	−0.14	0.44	0.08	0.24	.739		
Oppressive feeling chest							2.44	.096
Momentary	0.35	−0.29	1.04	0.05	0.29	.860		
Retrospective	2.00	1.00	0.31	−0.20	0.22	.497		
	**SSQ Fatigue**							
	Decline (*N* = 8)	Same (*N* = 7)	Improve (*N* = 11)					
Energetic							9.64	<.001*
Momentary	0.02	0.20	1.23	0.77	0.18	<.001*		
Retrospective	−1.50	0.86	0.91	−0.11	0.19	.581		
Tired							5.51	.007*
Momentary	0.07	0.61	1.31	0.50	0.19	.015*		
Retrospective	2.00	1.29	0.45	−0.23	0.22	.220		
	**SSQ Pain**							
	Decline (*N* = 3)	Same (*N* = 9)	Improve (*N* = 14)					
Pain chest							0.32	.813
Momentary	0.19	0.42	0.57	0.58	0.63	.373		
Retrospective	−0.33	−0.00	0.21	−0.08	0.30	.790		
Pain							0.09	.967
Momentary	−0.06	−0.08	−0.11	- < 0.01	0.42	.998		
Retrospective	0.67	0.44	2.00	0.40	0.29	.118		

Change scores of all negative items are reversed. A positive change score indicates an improvement in HRQOL, a negative change score indicates a decline in HRQoL. A positive β of the positive items = more positive mood associated with an improvement in mood. A positive β of the negative items = decline negative mood/symptomology associated with an improvement in health/mood. All regression models were adjusted for baseline momentary (mean) and retrospective scores

*P values < 0.05 were considered significant

If we found significant differences in associations between momentary and retrospective change with criterion measures of change, momentary items were always more strongly associated with the criterion measure than the retrospective items, and in the expected direction. *Momentary mood items* ‘cheerful’, ‘energetic’ and ‘happy’ were significantly associated with the criterion variable in the expected direction; feeling more energetic and happy was associated with better emotional state. *Retrospective mood item* ‘energetic’ was also significantly associated with the criterion variable, however, in the unexpected direction; feeling less energetic was associated with a better emotional state. *Momentary CAD symptom item* ‘shortness of breath’ was significantly associated with self-reported NYHA class in the expected direction; less shortness of breath was associated with improved physical functioning. No associations were found for any of the *retrospective CAD items*. *Momentary fatigue items* ‘energetic’ and ‘tired’ were significantly associated with the criterion variable in the expected direction; less momentary fatigue (feeling more energetic and less tired) was associated with an improvement in fatigue (feeling less tired). No significant associations were found for any of the *retrospective fatigue items*. For both the *momentary and retrospective pain items* we found no associations with the criterion variable of pain.

The hierarchical regression analyses showed that adding momentary HRQoL change to a model that included change in retrospective HRQoL to predict change according to the criterion measures, significantly improved the fit of the model. This was the case for most items where a change in HRQoL was significantly associated with the criterion measure of change in HRQoL. Only for the significant association between the momentary item ‘tired’ and NYHA class we did not find a significant improvement in fit (Table 2).

## Discussion

The current study indicates that momentary assessments yield a higher criterion validity in detecting changes in HRQoL following cardiac intervention than retrospective measures. This finding is, in part, surprising as it points to a paradox. Given that the criterion measures were retrospective in nature one could have expected these to yield stronger relationships with the retrospective HRQoL items than the momentary HRQoL items due to common method variance. On the other hand, retrospective change scores may be more biased than those based on the aggregated momentary scores. For example, when patients are asked to recall their average experience during the past week, retrospective scores may be disproportionately influenced by the most extreme experience (“peak effect”) or the most recent experience (“recency effect”) [23]. Our study was conducted among cardiac patients with comorbidities. Possibly, patients with comorbidities, in particular, experience substantial fluctuations in their daily HRQoL.

The choice of the retrospective criterion measures may be criticized as they would a priori benefit the retrospective HRQoL measures. However, as much as we would have wanted to administer momentary criterion measures, they were not available. We chose the criterion measures that were relevant to clinical practice. The NYHA class is a common measure used by clinicians to assess their patient’s performance status, similar to the Karnofsky Performance Status scale for oncology [24] or Eastern Cooperative Oncology Group [25]. The SSQ can be seen as a formalization of the question clinicians ask their patients in subsequent consultations; “how have you been doing since I last saw you?” Not surprisingly, this criterion measure had been proposed by an oncologist [21].

Interestingly, the momentary items formed the expected scale structure with adequate reliability on both baseline and follow-up. Conversely, none of the expected scales were found for the retrospective items at either of the measurement points. We had deliberately devised items for EMA application and had adapted these for retrospective use. Such simple items (“The past week I felt tired”, “The past week I felt happy”) are not uncommon in retrospective HRQoL questionnaires. Therefore, we did not expect them to perform poorly from a psychometric perspective. Most likely this was due to our sample size which is small for retrospective HRQoL measures. The small sample size likely resulted in a larger error variance for the retrospective items than for the momentary items because the latter were based on the average of multiple assessments.

Despite the higher criterion validity of momentary assessments, we would like to emphasize that both momentary and retrospective measures are needed as they provide unique, valuable, and complementary information about patient’s HRQoL. EMA is needed when the objective is to gain insight into daily fluctuations of patients’ momentary experiences in their natural environment. Retrospective measures are particularly required when one aims to assess patients’ global evaluation of their health and quality of life. Clearly, HRQoL comprises both types of experiences. Given that each assessment method also has its own advantages and disadvantages, decisions to use one method versus another should be guided by the research questions, study design and population. For example, momentary assessments may be less feasible in studies which require patients with little experience in using smartphones, and/or who are easily burdened due to a poor health condition. For those patients, standard questionnaires may be more appropriate.

### Limitations

Several limitations of this study should be noted. First, the sample size of 26 is small for HRQoL studies. However, it is common for intensive EMA studies, requiring respondents to complete a number of questions multiple times a day for a number of consecutive days. Second, our sample consisted of patients with CAD who received CABG or PCI, most of whom were older males. These results may therefore not generalize to patients with other sociodemographic characteristics, diseases, or types of interventions. Third, we did not adopt a minimum completion rate for participants’ momentary data to be eligible. We used the aggregated mean scores as these remain the best possible estimator for each subject, thereby acknowledging that the ‘error’ divergence from the true subject score becomes smaller when the number of assessments within subjects increase. Fourth, the use of change scores has been criticized for their unreliability [26]. Change scores tend to have greater measurement error than the original scores and they tend to have moderate negative correlations with baseline scores. However, change scores are commonly used in clinical studies. Fifth, as a consequence of poor psychometric performance of the retrospective items, all analyses were performed at item-level. It is unknown whether the same results would hold when multi-item composite scores could be used. Finally, as a consequence of the analysis at item-level, we needed to conduct a relatively large number of analyses in relation to the sample size. Whereas individual results may be less informative, a clear, overall pattern emerged, favoring consistently the momentary assessments over the retrospective ones.

### Strengths

This is the first study to our knowledge that administered EMA data for two separate one-week assessment periods before and after cardiac intervention. Adherence to the two 1-week assessment periods of EMA was good; patients answered on average 76% of the beeps, which is similar to other studies investigating momentary HRQoL [9]. Further, the time period of the EMA coincides with the one-week time frame of the retrospective measures. Also, we selected a patient group suitable for assessing changes in HRQoL, as HRQoL increases following cardiac revascularization procedures. Finally, all patients received an iPod for the duration of the assessment periods to assess the EMA questionnaire, to avoid a potential selection bias when only patients in the possession of a smart phone would be included.

## Conclusion

Momentary assessments were found to yield a higher criterion validity in detecting changes in HRQoL following cardiac revascularization than traditional retrospective measures. These results are encouraging and call for more research investigating the validity and reliability of EMA measures to expand our armamentarium of psychometrically sound HRQoL measures.

## Supplementary Information

**Additional file 1: Table S1.** Number of completed and missing responses for each assessment period. **Table S2.** Means and standard deviations of momentary and retrospective HRQoL items at baseline and follow-up. **Table S3.** Cronbach’s alpha coefficients of momentary and retrospective HRQoL scales at baseline and follow-up. **Table S4**. Results of exploratory factor analysis of momentary HRQoL items at baseline. **Table S5.** Results of exploratory factor analysis of momentary HRQoL items at follow-up. **Table S6.** Results of exploratory factor analysis of the retrospective HRQoL items at baseline. **Table S7.** Results of exploratory factor analysis of the retrospective HRQoL items at follow-up.

**Additional file 2.**

## Data Availability

Minimal dataset will be made available as supplementary materials.

## Abbreviations

HRQoL: Health-related quality of life
EMA: Ecological momentary assessment
CAD: Coronary artery disease
CABG: Coronary artery bypass graft
PCI: Percutaneous coronary intervention
NYHA class: New York Heart Association functioning classification system
SSQ: Subjective Significance Questionnaire
EFA: Exploratory factor analysis
