# Supplementary material for: Ecological momentary assessment versus retrospective assessment for measuring change in health-related quality of life following cardiac intervention
Source: J Patient Rep Outcomes. 2020 Nov 16;4:98. doi: 10.1186/s41687-020-00261-2 (PMC7669938; doi:10.1186/s41687-020-00261-2)
Supplement: Supplementary file 1 — Additional file 1: Table S1. Number of completed and missing responses for each assessment period. Table S2. Means and standard deviations of momentary and retrospective HRQoL items at baseline and follow-up. Table S3. Cronbach’s alpha coefficients of momentary and retrospective HRQoL scales at baseline and follow-up. Table S4. Results of exploratory factor analysis of momentary HRQoL items at baseline. Table S5. Results of exploratory factor analysis of momentary HRQoL items at follow-up. Table S6. Results of exploratory factor analysis of the retrospective HRQoL items at baseline. Table S7. Results of exploratory factor analysis of the retrospective HRQoL items at follow-up. [file 41687_2020_261_MOESM1_ESM.docx]

**SUPPLEMENTS**

**Table S1.** Number of completed and missing responses for each assessment period.

|  | **Completed** | **Missing (momentary, retrospective)** |
| --- | --- | --- |
| Baseline | 31 | 6 (4,2) |
| Follow-up T2 | 23 | 5 (3,2) |
| Follow-up T3 | 3 | 0 (0,0) |
| Baseline + follow-up | 26 | 11 (7,4) |

**Table S2.** Means and standard deviations of momentary and retrospective HRQoL items at baseline and follow-up.

| **HRQoL items** | **Mean baseline (SD)** | **Mean follow-up (SD)** | **Mean change (SD)** |
| --- | --- | --- | --- |
| Cheerful |  |  |  |
| Momentary | 4.78 (1.27) | 5.06 (1.22) | 0.28 (0.91) |
| Retrospective | 3.04 (1.93) | 3.69 (1.95) | 0.65 (2.45) |
| Relaxed |  |  |  |
| Momentary | 4.74 (1.22) | 5.19 (1.09) | 0.45 (0.86) |
| Retrospective | 2.62 (1.72) | 3.85 (1.74) | 1.23 (1.77) |
| Energetic |  |  |  |
| Momentary | 3.96 (1.35) | 4.55 (1.37) | 0.58 (0.93) |
| Retrospective | 3.15 (1.93) | 3.31 (2.41) | 0.15 (2.89) |
| Happy |  |  |  |
| Momentary | 5.01 (1.34) | 5.42 (1.77) | 0.41 (0.98) |
| Retrospective | 3.65(1.98) | 3.46 (2.28) | -0.19 (2.87) |
| Anxious |  |  |  |
| Momentary | 1.85 (1.41) | 1.49 (0.99) | 0.36 (0.95) |
| Retrospective | 3.38 (2.10) | 3.62 (2.17) | -0.23 (2.60) |
| Sad |  |  |  |
| Momentary | 1.77 (1.30) | 1.56 (1.05) | 0.22 (0.91) |
| Retrospective | 3.15 (2.13) | 3.19 (2.19) | -0.04 (2.55) |
| Irritated |  |  |  |
| Momentary | 1.66 (1.17) | 1.52 (0.73) | 0.14 (0.90) |
| Retrospective | 3.23 (2.10) | 3.88 (2.07) | -0.65 (2.28) |
| Worried |  |  |  |
| Momentary | 1.85 (1.47) | 1.52 (1.03) | 0.33 (0.83) |
| Retrospective | 3.27 (1.82) | 2.96 (2.41) | 0.31 (2.20) |
| Pain chest |  |  |  |
| Momentary | 1.86 (1.57) | 1.39 (0.86) | 0.48 (1.05) |
| Retrospective | 3.42 (1.94) | 3.35 (1.92) | 0.08 (2.64) |
| Shortness of breath |  |  |  |
| Momentary | 2.89 (1.70) | 2.23 (1.67) | 0.66 (1.85) |
| Retrospective | 3.00 (1.83) | 2.31 (1.76) | 0.69 (2.22) |
| Tight feeling chest |  |  |  |
| Momentary | 2.24 (1.66) | 1.64 (1.03) | 0.60 (1.15) |
| Retrospective | 3.23 (2.08) | 3.15 (2.13) | 0.08 (2.02) |
| Oppressive feeling chest |  |  |  |
| Momentary | 2.28 (1.62) | 1.67 (1.08) | 0.60 (1.18) |
| Retrospective | 4.04 (1.95) | 3.35 (2.04) | 0.69 (2.00) |
| Tired |  |  |  |
| Momentary | 3.51 (1.81) | 2.77 (1.73) | 0.74 (1.30) |
| Retrospective | 3.31 (1.93) | 2.15 (1.91) | 1.15 (1.91) |
| Pain |  |  |  |
| Momentary | 1.90 (1.56) | 2.00 (1.72) | -0.09 (0.69) |
| Retrospective | 3.65 (2.00) | 2.35 (1.74) | 1.31 (2.05) |
| **Criterion measures HRQoL change** | **Mean baseline (SD)** | **Mean follow-up (SD)** | **Mean change (SD)** |
| NYHA class | 2.42 (1.03) | 1.69 (0.97) | 0.73 (1.19) |
| SSQ Mental | N/A | 4.27 (1.43) | N/A |
| SSQ Fatigue | N/A | 4.31 (1.74) | N/A |
| SSQ Pain | N/A | 5.19 (1.47) | N/A |

*_Note_*_. Change scores of the negative items are reversed, such that a_ *_positive change score_* _indicates an_ *_improvement health_* _and a_ *_negative change score_* _indicates a_ *_decline in health. SSQ_*_>4 indicates an improvement HRQoL;_ *_SSQ_*_==4 indicates no improvement/worsening HRQoL;_ *_SSQ_*_<4 indicates a worsening in HRQoL._ *_Change in NYHA class_*_>0 indicates an improvement in physical functioning;_ *_change in NYHA class_* _== 0 indicates no improvement/worsening physical functioning;_ *_change in NYHA class_*_<0 indicates a worsening in physical functioning_*_._* *_No p-values for the difference scores_* _were calculated because our objective was not to test the change in HRQoL before and after cardiac intervention._

**Table S3.** Cronbach’s alpha coefficients of momentary and retrospective HRQoL scales at baseline and follow-up

|  | **Baseline** | | **Follow-up** | |
| --- | --- | --- | --- | --- |
| **HRQoL scales** | **Momentary** | **Retrospective** | **Momentary** | **Retrospective** |
| Positive mood | 0.95 | 0.53 | 0.96 | 0.40 |
| Negative mood | 0.94 | 0.64 | 0.94 | 0.79 |
| CAD symptoms | 0.96 | 0.66 | 0.86 | 0.86 |
| Fatigue | 0.88 | 0.59 | 0.90 | 0.27 |
| Pain | 0.69 | 0.53 | 0.63 | 0.71 |

**Table S4**. Results of exploratory factor analysis of momentary HRQoL items at baseline

| **HRQoL items** | **Factor loadings** | | | | |
| --- | --- | --- | --- | --- | --- |
|  | **Factor 1** | **Factor 2** | **Factor 3** | **Factor 4** | **Factor 5** |
| Positive Mood |  |  |  |  |  |
| Cheerful | **-0.34** | **-0.46** | **-0.53** | **-0.55** | -0.22 |
| Relaxed | **-0.36** | **-0.30** | **-0.56** | **-0.56** | **-0.40** |
| Energetic | -0.21 | **-0.31** | **-0.89** | -0.27 | -0.02 |
| Happy | -0.27 | **-0.44** | **-0.33** | **-0.74** | -0.02 |
| Negative Mood |  |  |  |  |  |
| Anxious | **0.32** | **0.61** | **0.55** | 0.20 | **0.36** |
| Sad | **0.40** | **0.79** | **0.36** | 0.25 | 0.18 |
| Irritated | **0.52** | **0.47** | 0.19 | **0.30** | 0.20 |
| Worried | **0.47** | **0.73** | 0.25 | **0.31** | -0.01 |
| CAD symptoms |  |  |  |  |  |
| Tired | **0.54** | **0.30** | **0.60** | 0.26 | 0.03 |
| Shortness of breath | **0.78** | 0.13 | **0.46** | 0.07 | 0.11 |
| Pain chest | **0.86** | **0.35** | 0.01 | 0.29 | 0.02 |
| Tightness chest | **0.88** | **0.31** | 0.26 | 0.22 | 0.03 |
| Oppressive chest | **0.84** | **0.33** | **0.30** | 0.17 | 0.13 |
| Pain |  |  |  |  |  |
| Pain | 0.24 | **0.56** | 0.25 | **0.40** | -0.06 |
| Pain chest | **0.86** | **0.35** | 0.01 | 0.29 | 0.02 |
| Fatigue |  |  |  |  |  |
| Tired | **0.54** | **0.30** | **0.60** | 0.26 | 0.03 |
| Energetic | -0.21 | **-0.31** | **-0.89** | -0.27 | -0.02 |
| **Explained Variance** | 0.31 | 0.22 | 0.20 | 0.14 | 0.03 |
| **Cumulative** | 0.31 | 0.53 | 0.70 | 0.84 | 0.87 |

*_Note_*_. Loadings >0.3 are in bold letters._

**Table S5.** Results of exploratory factor analysis of momentary HRQoL items at follow-up.

| **HRQoL items** | **Factor loadings** | | | | |
| --- | --- | --- | --- | --- | --- |
|  | **Factor 1** | **Factor 2** | **Factor 3** | **Factor 4** | **Factor 5** |
| Positive Mood |  |  |  |  |  |
| Cheerful | **-0.85** | **-0.43** | -0.26 | 0.02 | 0.15 |
| Relaxed | **-0.81** | -0.25 | **-0.32** | -0.09 | 0.01 |
| Energetic | **-0.93** | -0.19 | -0.18 | 0.06 | 0.04 |
| Happy | **-0.74** | **-0.38** | -0.26 | -0.14 | 0.20 |
| Negative Mood |  |  |  |  |  |
| Anxious | **0.33** | **0.85** | 0.27 | 0.10 | -0.02 |
| Sad | **0.38** | **0.86** | 0.28 | 0.10 | 0.06 |
| Irritated | **0.32** | 0.26 | **0.69** | **0.60** | 0.05 |
| Worried | 0.22 | **0.91** | **0.30** | 0.14 | -0.05 |
| CAD symptoms |  |  |  |  |  |
| Tired | **0.81** | 0.17 | **0.30** | 0.13 | **0.33** |
| Shortness of breath | **0.80** | 0.26 | 0.12 | 0.16 | **0.36** |
| Pain chest | 0.16 | **0.40** | **0.71** | 0.17 | 0.09 |
| Tightness chest | **0.33** | 0.21 | **0.86** | 0.06 | -0.01 |
| Oppressive chest | 0.29 | **0.44** | **0.85** | -0.09 | -0.01 |
| Pain |  |  |  |  |  |
| Pain | 0.25 | **0.63** | **0.37** | -0.17 | 0.22 |
| Pain chest | 0.16 | **0.40** | **0.71** | 0.17 | 0.09 |
| Fatigue |  |  |  |  |  |
| Tired | **0.81** | 0.17 | **0.30** | 0.13 | **0.33** |
| Energetic | **-0.93** | -0.19 | -0.18 | 0.06 | 0.04 |
| **Explained Variance** | 0.34 | 0.26 | 0.23 | 0.04 | 0.02 |

*_Note_*_. Loadings >0.3 are in bold letters._

**Table S6.** Results of exploratory factor analysis of the retrospective HRQoL items at baseline.

| **HRQoL items** | **Factor loadings** | | | | |
| --- | --- | --- | --- | --- | --- |
|  | **Factor 1** | **Factor 2** | **Factor 3** | **Factor 4** | **Factor 5** |
| Positive Mood |  |  |  |  |  |
| Cheerful | 0.20 | 0.24 | -0.02 | 0.10 | **0.94** |
| Relaxed | 0.28 | **0.64** | 0.13 | 0.03 | 0.23 |
| Energetic | **0.43** | 0.20 | 0.07 | **0.53** | -0.01 |
| Happy | 0.18 | 0.28 | **0.54** | 0.29 | -0.16 |
| Negative Mood |  |  |  |  |  |
| Anxious | -0.09 | **0.42** | **0.44** | -0.03 | **0.33** |
| Sad | **0.75** | **0.49** | 0.17 | 0.06 | 0.01 |
| Irritated | **0.47** | -0.05 | 0.16 | 0.28 | 0.15 |
| Worried | **0.31** | **0.47** | **0.38** | 0.14 | 0.11 |
| CAD symptoms |  |  |  |  |  |
| Tired | 0.07 | **0.80** | 0.11 | **0.30** | 0.06 |
| Shortness of breath | 0.04 | 0.19 | 0.29 | **0.94** | 0.11 |
| Pain chest | **0.46** | 0.12 | 0.04 | 0.11 | 0.06 |
| Tightness chest | **0.41** | **0.33** | **0.69** | 0.04 | 0.08 |
| Oppressive chest | 0.16 | 0.00 | **0.66** | 0.21 | 0.00 |
| Pain |  |  |  |  |  |
| Pain | **0.65** | 0.05 | **0.37** | -0.09 | 0.06 |
| Pain chest | **0.46** | 0.12 | 0.04 | 0.11 | 0.06 |
| Fatigue |  |  |  |  |  |
| Tired | 0.07 | **0.80** | 0.11 | **0.30** | 0.06 |
| Energetic | **0.43** | 0.20 | 0.07 | **0.53** | -0.01 |
| **Explained Variance** | 0.15 | 0.15 | 0.13 | 0.11 | 0.08 |

*_Note_*_. Loadings >0.3 are in bold letters._

**Table S7.** Results of exploratory factor analysis of the retrospective HRQoL items at follow-up.

| **HRQoL items** | **Factor loadings** | | | | |
| --- | --- | --- | --- | --- | --- |
|  | **Factor 1** | **Factor 2** | **Factor 3** | **Factor 4** | **Factor 5** |
| Positive Mood |  |  |  |  |  |
| Cheerful | 0.02 | 0.09 | -0.07 | **0.86** | -0.05 |
| Relaxed | 0.09 | **0.56** | **0.41** | **0.43** | 0.22 |
| Energetic | 0.10 | **0.45** | **0.44** | -0.10 | 0.06 |
| Happy | 0.03 | 0.01 | 0.21 | -0.01 | **0.73** |
| Negative Mood |  |  |  |  |  |
| Anxious | 0.07 | 0.24 | **0.70** | -0.06 | **0.44** |
| Sad | **0.40** | **0.89** | 0.09 | 0.12 | -0.03 |
| Irritated | 0.14 | 0.12 | **0.93** | 0.06 | 0.11 |
| Worried | **0.54** | **0.75** | 0.23 | 0.13 | 0.05 |
| CAD symptoms |  |  |  |  |  |
| Tired | **0.68** | **0.38** | 0.08 | 0.21 | 0.03 |
| Shortness of breath | **0.84** | 0.23 | 0.26 | -0.05 | -0.24 |
| Pain chest | **0.68** | 0.15 | 0.25 | 0.05 | 0.27 |
| Tightness chest | **0.54** | **0.45** | 0.24 | **0.38** | 0.19 |
| Oppressive chest | **0.75** | 0.08 | -0.22 | -0.11 | 0.10 |
| Pain |  |  |  |  |  |
| Pain | **0.64** | **0.49** | 0.28 | 0.13 | -0.23 |
| Pain chest | **0.68** | 0.15 | 0.25 | 0.05 | 0.27 |
| Fatigue |  |  |  |  |  |
| Tired | **0.68** | **0.38** | 0.08 | 0.21 | 0.03 |
| Energetic | 0.10 | **0.45** | **0.44** | -0.10 | 0.06 |
| **Explained Variance** | 0.24 | 0.19 | 0.15 | 0.09 | 0.07 |

*_Note_*_. Loadings >0.3 are in bold letters._
